# A Data-Driven Preprocessing Framework for Atrial Fibrillation Intracardiac Electrocardiogram Analysis

**DOI:** 10.3390/e25020332

**Published:** 2023-02-10

**Authors:** Xiangzhen Kong, Vasanth Ravikumar, Siva K. Mulpuru, Henri Roukoz, Elena G. Tolkacheva

**Affiliations:** 1 Department of Electrical and Computer Engineering, University of Minnesota, Minneapolis, MN 55455, USA; 2 Division of Cardiovascular Diseases, Mayo Clinic, Rochester, MN 55905, USA; 3 Division of Cardiology, University of Minnesota, Minneapolis, MN 55455, USA; 4 Department of Biomedical Engineering, University of Minnesota, Minneapolis, MN 55455, USA; 5 Lillehei Heart Institute, University of Minnesota, Minneapolis, MN 55455, USA; 6 Institute for Engineering in Medicine, University of Minnesota, Minneapolis, MN 55455, USA

**Keywords:** intracardiac electrograms, atrial fibrillation, catheter ablation, multiscale frequency, bandpass filter, DBSCAN, Pearson’s correlation, earth mover’s distance

## Abstract

Atrial Fibrillation (AF) is the most common cardiac arrhythmia. Signal-processing approaches are widely used for the analysis of intracardiac electrograms (iEGMs), which are collected during catheter ablation from patients with AF. In order to identify possible targets for ablation therapy, dominant frequency (DF) is widely used and incorporated in electroanatomical mapping systems. Recently, a more robust measure, multiscale frequency (MSF), for iEGM data analysis was adopted and validated. However, before completing any iEGM analysis, a suitable bandpass (BP) filter must be applied to remove noise. Currently, no clear guidelines for BP filter characteristics exist. The lower bound of the BP filter is usually set to 3–5 Hz, while the upper bound (${\bar{BP}}^{th}$) of the BP filter varies from 15 Hz to 50 Hz according to many researchers. This large range of ${\bar{BP}}^{th}$ subsequently affects the efficiency of further analysis. In this paper, we aimed to develop a data-driven preprocessing framework for iEGM analysis, and validate it based on DF and MSF techniques. To achieve this goal, we optimized the ${\bar{BP}}^{th}\;$ using a data-driven approach (DBSCAN clustering) and demonstrated the effects of different ${\bar{BP}}^{th}$ on subsequent DF and MSF analysis of clinically recorded iEGMs from patients with AF. Our results demonstrated that our preprocessing framework with ${\bar{BP}}^{th}$ = 15 Hz has the best performance in terms of the highest Dunn index. We further demonstrated that the removal of noisy and contact-loss leads is necessary for performing correct data iEGMs data analysis.

## 1. Introduction

Atrial Fibrillation (AF) is the most common cardiac arrhythmia. It is associated with an increased risk of heart failure, dementia, and stroke [1,2]. According to the estimation in 2014, 2.7 to 6.1 million people in the United States are afflicted by AF [3]. Catheter ablation, which has proved to be an efficient solution in most of the patients suffering from AF, is a minimally invasive procedure where clinicians ablate abnormal electrical pathways inside the heart tissue [4,5]. During the ablation procedure, intracardiac electrograms (iEGMs) are simultaneously collected from the electroanatomical mapping systems and analyzed to guide the ablation. The iEGMs are the recordings of local electrical activities within the various chambers of the heart by using multipolar electrodes placed inside the heart. iEGMs provide real-time information regarding the state of the cardiac tissue surrounded by the catheter tip and thus promote real-time analysis and future investigations [6,7]. To investigate important diagnostic information, the preprocessing of iEGMs, which has a profound effect on further analysis, needs to be addressed in terms of the characteristics of different biomedical signals and various analysis tools [8,9]. In this research, we aim to determine a data-driven preprocessing framework of iEGMs for subsequent AF analysis, especially for frequency analysis such as dominant frequency (DF) and multiscale frequency (MSF).

As a classical method for iEGM preprocessing, Botteron’s approach, includes the following steps: (1) bandpass filtering at 40–250 Hz; (2) rectification; and (3) lowpass filtering at 20 Hz [10,11]. It eliminates the signal content within the beat-to-beat interval lower than 40 Hz and restores the low frequency components by using rectification and lowpass filtering. However, the transformed and simplified signals lose morphological information regarding the atrial beat and can create unrelated frequency components under some observations [12,13]. Some of these eliminated features may be related to wavefront propagation. In addition, the negative portion of an iEGM is not always caused by far-field noise but is removed by rectification, thus making it unreliable. Therefore, many studies preprocess raw iEGM signals with only bandpass (BP) filtering [6,14,15]. Specifically, in a previous study [16,17], the authors used an IIR Butterworth bandpass filter to smooth iEGMs at frequencies of 0.5–60 Hz or 0–60 Hz. In another study [18], the iEGMs were firstly bandpass-filtered at 30 Hz to 400 Hz and then filtered at 3 Hz to 15 Hz. The authors of [19] tapered the signal at the edges to a 0 value by using the Hanning window and then rectified and processed them with a nonbiased 3–15 Hz IIR bandpass filter. A fourth-order Butterworth filter was applied to the summed data in another study [20]. In our group, we also applied a 3–30 Hz third-order IIR Butterworth BP filter in previous iEGM studies [21,22,23]. IIR Butterworth BP filters with different orders can also be seen in similar biomedical signal analyses [24,25,26].

Preprocessing of iEGMs has several steps that are crucial and affect the overall analysis results. First, it is important to identify all outlier signals, such as white noise, motion artifact noise, or no signal due to contact-loss leads, which might have a negative effect on further analysis. However, it is still unclear whether removing these noisy and contact-loss signals from the original clinical iEGM is a necessary preprocessing step. Second, while the BP filter is typically used as a default preprocessing step, the guidelines for BP characteristics are unclear. While several studies have indicated that the lower bound of the BP filter should be between 3 Hz and 5 Hz, the guidelines for the upper bound (${\bar{BP}}^{th}$) still have a large range, which varies from 15 Hz to 50 Hz or even 250 Hz to 300 Hz [6,14]. However, the accuracy of all approaches for iEGM analysis are sensitive to the initial filtering, including DF [27,28], MSF [29,30,31], local activation time [32], complex fractionated atrial electrograms [33], and nonlinear analysis [18,34,35]. One theoretical study [36] indicated ${\bar{BP}}^{th}$ = 15 Hz should be used during the harmonic component analysis. However, no practical assessment of BP filter parameters has been performed for iEGMs. On the contrary, other studies point towards 4–10 Hz or 4–12 Hz being the physiologically relevant range that frequency analysis should focus on [37,38,39]. Thus, direct comparisons of further analysis under different influences of initial filtering still need to be addressed. 

In this paper, we aim to build a data-driven framework to perform correct preprocessing of raw atria iEGMs signals from clinical procedures. This aims to further improve subsequent AF frequency analysis. Specifically, we determined characteristics of ideal and fifth-order IIR Butterworth BP filters by using density-based spatial clustering of applications with noise (DBSCAN) and evaluated the necessity of removing noisy, and contact-loss iEGM leads from the original iEGMs prior to analysis. We further demonstrated the application of this framework for subsequent DF and MSF analysis of the iEGM signals. Finally, we showed the identification of abnormal electrical sites using the preprocessing steps proposed in AF patients.

## 2. Materials and Methods

### 2.1. The Clinical iEGMs from Patients with AF

Bipolar iEGMs obtained from the left atria (LA) were collected during an electrophysiological study performed at the Mayo Clinic (Rochester, MN) *n* = 1 and University of Minnesota (Minneapolis, MN) *n* = 8, with prior approval from the University of Minnesota’s Institutional Review Board (IRB)-approved protocol #STUDY00003128. All experiments were performed in accordance with relevant guidelines and regulations. The iEGM datasets were collected retrospectively. Simultaneous iEGM collection was carried out by using the CARTO (Biosense Webster, CA) system, which has a sensor position accuracy of 0.8 mm and 5°. Bipolar iEGMs (N = 10) were recorded at a different number of spatial sites using a multielectrode, contact-based catheter (PENTARAY™ Catheter, Biosense Webster, Irvine, CA, USA). These were evenly distributed across the LA at a sample rate of 977 Hz for all iEGMs and a variable duration of 5 to 15 s. No downsampling or upsampling was performed.

The starting and ending points of iEGMs during the mapping step were noted by clinicians during the 4–8 h ablation procedure. Next, three researchers independently performed iEGM cleaning, and only reserved channels indicated by all team members were used for further analysis. Signals with extremely low amplitude, long gap, high noise corruption, loss of contact, low signal-to-noise ratio (SNR), or white noise were all removed. After cleaning, 5–10 channels were reserved for each spatial location. Finally, two sets of iEGMs for each patient (*n* = 9) were created (see Table 1): all iEGMs (Set 1) and clean iEGMs (Set 2).

The data from Patient 1 at Mayo Clinic is used as a representative example to introduce our approach. iEGMs in this dataset were collected at M = 20 different spatial sites using N = 10 electrodes. After removing noisy and contact-loss leads, the total number of clean signals from Patient 1 was reduced from 200 to 141 (see Total_1_ and Total_2_ in Table 1). This illustrative example was provided by clinicians.

### 2.2. BP Filtering and DF/MSF Analysis

iEGMs from Table 1 (both Sets 1 and 2) were filtered using BP filters with a lower bound of 3 Hz and different upper bounds: ${\bar{BP}}^{th}$ = 10 Hz, 13 Hz, 15 Hz, 17 Hz, 20 Hz, 25 Hz, and 30 Hz. The ideal BP filter was designed such that all the magnitudes lying within the passband of the filter are retained with a gain of 1 and the stopbands were set to 0 in the frequency domain. This approach was used to retain all the frequency components with maximum possible resolution despite the disadvantages of having high spectral leakage. To illustrate the importance and practical implementation, we also include a fifth-order IIR Butterworth BP filter, which has commonly been used in many previous studies [14,15,16,17,18,19,20,21,22,23].

To demonstrate the results of the different filtering characteristics of subsequent iEGM analysis, two frequency-based approaches were used: DF [27,28] and MSF [29,30,31]. DF is a traditional technique that is widely used in many clinical applications [40,41]. However, while effective for stationary signals, DF loses robustness when analyzing more chaotic signals due to noise, misleading phase, and activation times that cause distortion [42]. As a more robust frequency approach, MSF uses eight log-Gabor filters with a relative filter bandwidth, one octave apart and thus can account for the contributions from various frequency components other than the DF. For instance, in our previous study, we demonstrated MSF has a more robust performance than DF to identify the driver of AF using ex vivo optical mapping experiments and numerical simulations [23]. In an ideal case, the DF and MSF converge where the spectrum has a single peak. Therefore, in practical cases, there is insignificant difference, as only the peaks close to the DF are considered in MSF. Details of these two methods are shown in Supplementary Materials.

Two matrices, ***DF*** and ***MSF*** of dimension N × M (10 × 20 here), were generated using iEGMs of Patient 1 from Set 1 for both DF and MSF for all 7 ${\bar{BP}}^{th}$:

(1) $$DF=\left[\begin{array}{ccc} df_{1,1} & \cdots & df_{1,M}\\ \vdots & \ddots & \vdots \\ df_{N,1} & \cdots & df_{N,M} \end{array}\right]$$

(2) $$MSF=\left[\begin{array}{ccc} msf_{1,1} & \cdots & msf_{1,M}\\ \vdots & \ddots & \vdots \\ msf_{N,1} & \cdots & msf_{N,M} \end{array}\right]$$

Similar ***DF*** and ***MSF*** matrices were separately obtained for Set 2.

### 2.3. Statistical Analysis

The mean (<DF> or <MSF>) and standard deviation (SD) of all rows (i.e., spatial sites) in the DF and MSF matrix (Equations (1) and (2)) at each ${\bar{BP}}^{th}$ for each patient were calculated. To illustrate the effect of ${\bar{BP}}^{th}$ across patients, these values were then averaged across all patients and spatial sites to get an overall mean <DF> and <MSF> of all iEGMs at each ${\bar{BP}}^{th}$. Similarly, the overall SD of <DF> and <MSF> at each ${\bar{BP}}^{th}$ was obtained. To show statistical significance, a one-way ANOVA test was performed, and the *p*-value threshold was set to 0.05 in this research (indicated as *). All the calculations and analyses were performed using custom-written MATLAB (MathWorks, Inc., Natick, MA, USA) scripts.

### 2.4. Correlation Analysis

To demonstrate whether efficiency of DF and MSF analysis are different between Set 1 and 2, two correlation approaches were used to measure the similarity.

Pearson’s correlation [34] is the covariance of the two variables divided by the product of their SDs. It quantifies the similarity between the efficiency of DF and MSF analysis at a given spatial site with the following calculation:

(3) $$r_{m}\left(DF,MSF\right)=\frac{\mathrm{cov}(\bar{df_{m}},\bar{msf_{m}})}{\sigma_{\bar{df_{m}}}\sigma_{\bar{msf_{m}}}}$$

where m = (1, 2, …, M) is the spatial site, $\bar{df_{m}}$ and $\bar{msf_{m}}$ are the m^th^ column of matrix ***DF*** and ***MSF*** from Equations (1) and (2), respectively. $\sigma_{\bar{df_{m}}}$ and $\sigma_{\bar{msf_{m}}}$ are the SD of all the elements in vector $\bar{df_{m}}$ and $\bar{msf_{m}}$. For the Pearson’s correlation, according to Equation (3), a threshold of p^th^ = ±0.6 was chosen to distinguish between high (>|r^th^|) and low (<|r^th^|) correlation between DF and MSF [30].

Pearson’s correlation method is sensitive to outliers [30]. To overcome this limitation, another similarity measuring index, earth mover’s distance (EMD), was used. EMD is robust to the presence of outliers since it is capable of providing partial matches, and it is independent of the magnitude of DF and MSF values but rather considers the distance between the values of the two approaches. EMD was used to measure the dissimilarity between two multidimensional distributions [43]. At each spatial site m, we calculate EMD_m_ (***DF***, ***MSF***) as follows:

(4) $$EMD_{m}\left(DF,MSF\right)=\frac{\sum_{i=1}^{N}\sum_{j=1}^{N}f_{ij}d_{ij}}{\sum_{i=1}^{N}\sum_{j=1}^{N}f_{ij}}$$

where m = (1, 2, …, M), f_ij_ is the flowrate between $\bar{df_{m}}$ and $\bar{msf_{m}}$ and d_ij_ is the Euclidean distance between $\bar{df_{m}}$ and $\bar{msf_{m}}$. For EMD, according to Equation (4), a threshold of EMD^th^ = 0.2 was chosen, to distinguish between high (<EMD^th^) and low (>EMD^th^) correlation between DF and MSF [30].

### 2.5. DBSCAN and Dunn Index

Density-based spatial clustering of applications with noise (DBSCAN) [44,45] is widely used in applications and designed to cluster data of arbitrary shapes in the presence of outliers. In this research, DBSCAN was implemented to further investigate the natural cluster information in the DF and MSF data space under the influence of different BP filters. The Dunn index, which is defined as the lowest inter-cluster distance divided by the highest inter-cluster distance, was calculated to evaluate the performance of each BP filter [46,47]. We only applied DBSCAN clustering to Set 2, which has Total_2_ pairs of DF and MSF observations. 

Normalization for DF and MSF values from 0 to 1 was performed to restrict the data range. The two ***DF*** and ***MSF*** matrices from Equations (1) and (2) were reshaped into vectors as $\bar{DF}$ and $\bar{MSF}$, and the new observation matrix ***X*** was defined as $\left(\bar{DF},\;\bar{MSF}\right)$:

(5) $$\bar{DF}=\left[\begin{array}{c} df_{1,1}\\ \vdots \\ df_{N,M} \end{array}\right]\;\;\;\bar{MSF}=\left[\begin{array}{c} msf_{1,1}\\ \vdots \\ msf_{N,M} \end{array}\right]$$

(6) $$X=\left[\begin{array}{cc} df_{1,1} & msf_{1,1}\\ \vdots & \vdots \\ df_{N,M} & msf_{N,M} \end{array}\right]=\left[\begin{array}{c} x_{1}\\ \vdots \\ x_{{Total}_{2}} \end{array}\right]$$

DBSCAN starts by finding the points within a given distance (***Eps***) of every observation (each row in Equation (6)), then identifies the core points with more than a specific number of neighbors (***MinPts***). The core points on the neighbor graph were connected, and then noncore points were assigned to a nearby cluster if the cluster was within *Eps*; otherwise, it was assigned to the outliers [47]. The iterations continued until all observations in Equation (6) for each ${\bar{BP}}^{th}$ were reached. For two-dimensional data, the default value of ***MinPts*** = 4 was used here and ***Eps*** was computed from input data **X** using a k-nearest neighbor (k-NN) search [48]. 

In order to evaluate the performance under influence of different each ${\bar{BP}}^{th}$, the Dunn index (DI) was calculated for every clustering result. Let $C_{1},C_{2},C_{3},C_{4},\ldots ,C_{k}$ be the clusters of ***X:***

(7) $$DI\left(X\right)=\frac{min_{i\neq j}\left\{\delta (C_{i},C_{j})\right\}}{max_{1\leq l\leq k}\left\{\Delta \left(C_{l}\right)\right\}}$$

where $\delta (C_{i},C_{j})=min_{x\epsilon C_{i},y\epsilon C_{j}}d\left(x,y\right)$, $\Delta \left(C_{l}\right)=max_{x\epsilon C_{l},y\epsilon C_{l}}d\left(x,y\right)$, d: C × C → **R** is a function that measures the distance between objects.

## 3. Results

### 3.1. The Influence of BP Filtering on DF and MSF

#### 3.1.1. Comparison of MSF and DF Analyses of iEGMs

**Figure 1:**
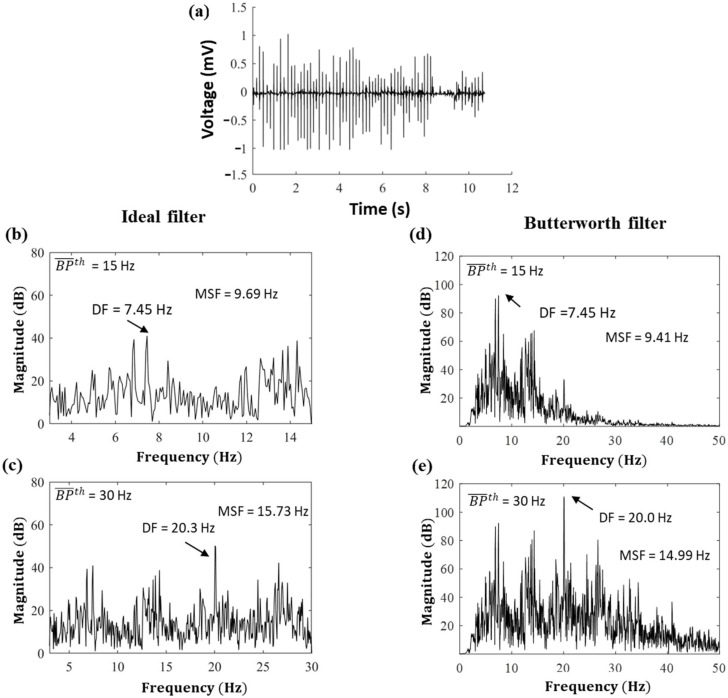
(**a**) A representative example of raw iEGMs recorded from Patient 1 Set 2. Corresponding power spectra from a single electrode after an ideal BP filtering with (**b**) ${\bar{BP}}^{th}$ = 15 Hz and (**c**) ${\bar{BP}}^{th}$ = 30 Hz; and IIR Butterworth BP filtering with (**d**) ${\bar{BP}}^{th}$ = 15 Hz and (**e**) ${\bar{BP}}^{th}$ = 30 Hz.

A representative example of raw iEGMs recorded from Patient 1 is shown in Figure 1a. The corresponding power spectra are shown in Figure 1b,c after an ideal BP filtering with ${\bar{BP}}^{th}$ = 15 Hz and ${\bar{BP}}^{th}$ = 30 Hz, and Figure 1d,e for the fifth-order Butterworth filter, respectively. Figure 1b,c shows that for the ideal BP filter, the value of DF is different from the corresponding MSF value in each power spectrum, and both DF and MSF values vary with ${\bar{BP}}^{th}$. Specifically, DF = 7.45 Hz, MSF = 9.69 Hz for ${\bar{BP}}^{th}$ = 15 Hz and DF = 20.03 Hz, MSF = 15.73 Hz for ${\bar{BP}}^{th}$ = 30 Hz. Similar results can also be observed for the case of the Butterworth filter in Figure 1d,e. For the example, DF = 7.45 Hz, MSF = 9.41 Hz for ${\bar{BP}}^{th}$ = 15 Hz and DF = 20.03 Hz, MSF = 14.99 Hz for ${\bar{BP}}^{th}$ = 30 Hz. Note that the DF values are the same both for the ideal and Butterworth filter, while MSF values are slightly different, most probably due to the difference in transition zone.

Figures S1 and S2 demonstrate MSF and DF values calculated from iEGMs of Patient 1 Set 2, from 20 spatial sites after applying ideal and Butterworth BP filters, respectively, with various ${\bar{BP}}^{th}$ ranging from 10 Hz to 30 Hz. Note that both DF and MSF increase as ${\bar{BP}}^{th}$ increases. Indeed, for the ideal BP filter, mean values of DF (<F> = 6.77 ± 0.927 Hz) and MSF (<MSF> = 7.07 ± 0.084 Hz) for ${\bar{BP}}^{th}$ = 10 Hz become <DF> = 12.84 ± 7.816 Hz and <MSF> = 17.76 +0.965 Hz, respectively, for ${\bar{BP}}^{th}$ = 30 Hz. In addition, changing ${\bar{BP}}^{th}$ significantly affects the overall distribution of DF—but not MSF—values. Specifically, DF and MSF are both clustering around their mean values for ${\bar{BP}}^{th}$ = 10 Hz. However, when ${\bar{BP}}^{th}$ is increased, DF has a wider distribution and diverges into upper and lower clusters, while MSF is still clustered around its mean. Similar trends were observed for the Butterworth BP filter (see Figure S2).

**Figure 2:**
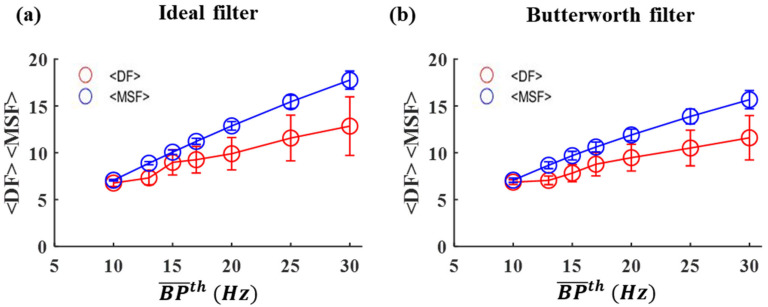
Mean of <DF> and <MSF> values and their standard deviations calculated from iEGMs of Patient 1, Set 2, from 20 spatial sites for various ${\bar{BP}}^{th}$ from (**a**) ideal and (**b**) Butterworth filters, respectively.

To further investigate the influence of ${\bar{BP}}^{th}$ on DF and MSF, <DF> and <MSF> across all spatial sites in Set 2 are shown in Figure 2a,b as a function of ${\bar{BP}}^{th}$. It was evident that both <DF> and <MSF> increase as ${\bar{BP}}^{th}$ increases. However, the SD of MSF is significantly different from DF and more compact at all values of ${\bar{BP}}^{th}$. For instance, the SD of MSF (0.965) is significantly smaller than the SD of DF (7.816, *p* < 0.05) at ${\bar{BP}}^{th}$ = 30 Hz. The same is true for all other ${\bar{BP}}^{th}\;$(*p* < 0.05 from one-way ANOVA). It can be noticed that MSF values are always clustered together (Figures S1 and S2), at any ${\bar{BP}}^{th}$, while the appearance of more clusters around <DF> is evident from Figures S1 and S2 as ${\bar{BP}}^{th}$ increases. These results clearly demonstrate that ${\bar{BP}}^{th}$ can affect the frequency analysis of iEGMs, and that DF is more sensitive to changes in ${\bar{BP}}^{th}$ as well as more variable than MSF. Moreover, the behavior of the ideal BP filter is very similar to the behavior of the Butterworth BP filter.

#### 3.1.2. Efficient ${\bar{BP}}^{th}$ Parameter Using DBSCAN

As was shown in Figures S1 and S2, the DF values of Patient 1 Set 2 diverge from <DF> as ${\bar{BP}}^{th}$ increases. Thus, there are several natural groups in the DF and MSF data space. To detect and investigate these clusters, the data-driven approach, DBSCAN, was implemented on paired DF–MSF data. Then, to identify the most efficient ${\bar{BP}}^{th}$, DI was calculated. The cluster formations corresponding to the example from Figure 2 are shown in Figures S3 and S4 for different ${\bar{BP}}^{th}$ values of ideal and Butterworth filters, respectively. Only one cluster was shown in Figures S3a and S4a. Then, two clusters appeared and remained at Figures S3b,c and S4c. A third cluster began presenting itself when increasing ${\bar{BP}}^{th}$ to 17 Hz and split into more clusters with complex information at ${\bar{BP}}^{th}$ = 30 Hz. It appears that clear separation between clusters and increased cluster compaction occurred at ${\bar{BP}}^{th}$ = 15 Hz (Figures S3c and S4c), which indicates a better clustering result. For other ${\bar{BP}}^{th}$, the clusters became less compact (more variance within one cluster). 

**Figure 3:**
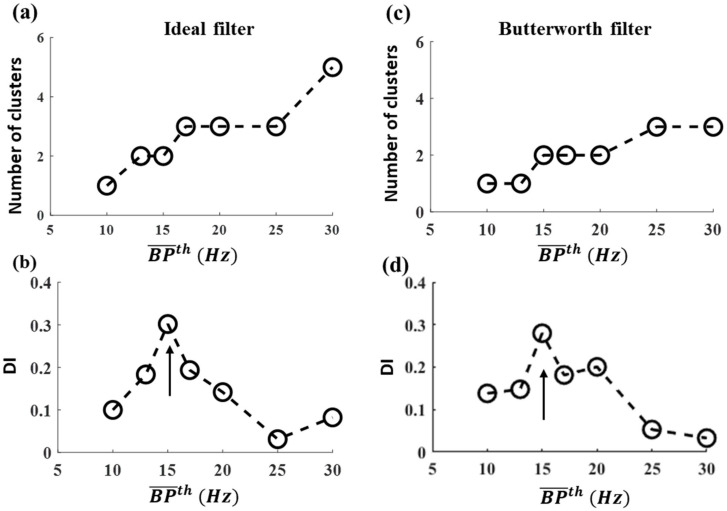
DBSCAN clustering results based on DF–MSF paired data calculated in Patient 1 Set 2 for ideal and Butterworth filters for different ${\bar{BP}}^{th\;}$: (**a**,**c**) number of clusters and (**b**,**d**) DI values. DI is maximum at ${\bar{BP}}^{th}$ = 15 Hz both for ideal and Butterworth filters, as indicated by an arrow.

The total number of clusters as a function of ${\bar{BP}}^{th}$ is shown in Figure 3a,c for the ideal and Butterworth filters, respectively. In order to identify the most efficient ${\bar{BP}}^{th}$ based on distributions for each clustering result shown in Figures S3 and S4, we calculated *DI.* The results are shown as a function of ${\bar{BP}}^{th}$ in Figure 3b,d for the ideal and Butterworth filters, respectively. Note that ${\bar{BP}}^{th}$ = 15 Hz (see arrow) had maximum DI over all ${\bar{BP}}^{th}$ values for both filters, which indicates that the clusters are compact. From 10 Hz to 15 Hz, the second cluster appeared, and DI increased to maximum. This observation suggests that some critical frequency components must exist between 10–15 Hz, which most probably corresponds to the physiological range for AF. Thus, 3–15 Hz is an efficient set of parameters to perform iEGM preprocessing of DF and MSF analysis for both ideal and IIR Butterworth filters. Therefore, for the rest of this manuscript, only the ideal filter is implemented.

#### 3.1.3. Efficient Preprocessing Framework on Clinical EGMs

In this section, the preprocessing framework developed in previous subsections was implemented in clinical EGM data. We applied it to Set 2, recorded from all (*n* = 9) patients with AF, and further implemented DF- and MSF-based frequency analysis. 

**Figure 4:**
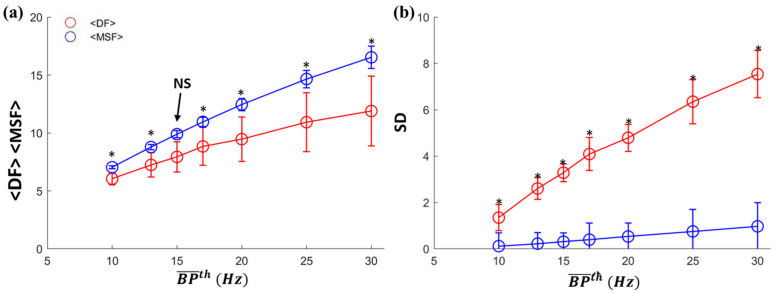
Mean <DF> and <MSF> (**a**) and their SD (**b**) of for all nine patients from Set 2 as a function of ${\bar{BP}}^{th}$. <DF> and <MSF> show only nonstatistical difference (*p* = 0.08) and work consistently at ${\bar{BP}}^{th}$ = 15 Hz. Statistic difference is indicated by the asterisk.

The distributions of <DF>, <MSF>, and their corresponding SDs from all patients are shown in Figure 4a,b, respectively, as a function of ${\bar{BP}}^{th}$. Note that <DF> and <MSF> across all patients increases with increasing ${\bar{BP}}^{th}$. <DF> and <MSF> are significantly different (indicated by *) at all ${\bar{BP}}^{th}$ except for ${\bar{BP}}^{th}$ = 15 Hz, indicating that only for this value of ${\bar{BP}}^{th}$, DF and MSF produce similar results. All *p* values are less than 0.05 except *p* = 0.083 for ${\bar{BP}}^{th}$ = 15 Hz. Compared with DF, the MSF technique has a more compact distribution in terms of constantly lower SD than DF (see Figure 4b). The SD of DF is always significantly higher than MSF regardless of ${\bar{BP}}^{th}$, as indicated by the asterisk.

**Figure 5:**
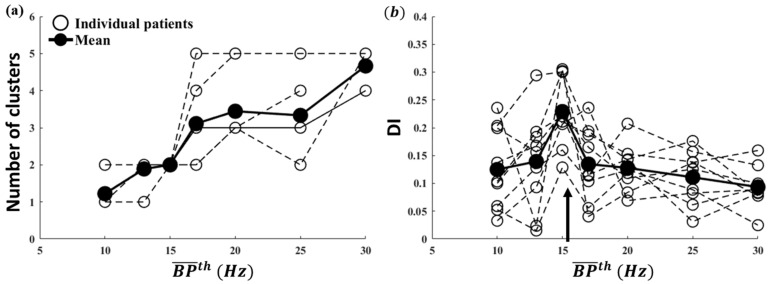
Averaged number of clusters (**a**) and DI (**b**) and among all nine patients in Set 2. The overall DI is still maximized at ${\bar{BP}}^{th}$ = 15 Hz, as indicated by an arrow in panel (**a**), and all patients showed two clear clusters at the same time at 15 Hz in panel (**b**).

Furthermore, we repeated DBSCAN clustering on DF and MSF analysis with different ${\bar{\;BP}}^{th}$ for Set 2 from each patient. The number of clusters and DI for each individual patient with the mean of all nine patients under different ${\bar{\;BP}}^{th}$ were calculated. The number of clusters from all nine patients as a function of ${\bar{BP}}^{th}$ is presented in Figure 5a. At ${\bar{BP}}^{th}$ = 15 Hz, all patients show two clear clusters with maximum DI. More clusters presented, beginning at ${\bar{BP}}^{th}$ = 17 Hz. Furthermore, the average DI and distribution of all nine patients (open circle) as a function of ${\bar{BP}}^{th}$ is shown in Figure 5b. Although two patients show higher DI at ${\bar{BP}}^{th}$ = 13 Hz and 17 Hz, respectively, the overall DI is still maximum at ${\bar{BP}}^{th}$ = 15 Hz, as shown by the thick curve. Thus, the 3–15 Hz BP filter is efficient and reasonable for DF and MSF analysis of iEGMs.

### 3.2. The Influence of Noisy and Contact-Loss Leads on DF and MSF Analysis

#### 3.2.1. Correlation Analysis

Pearson’s correlation and EMD were performed separately to quantify the differences between the Set 1 and Set 2 iEGM signals of Patient 1 to demonstrate the necessity of removing noisy and contact-loss leads in this case.

**Figure 6:**
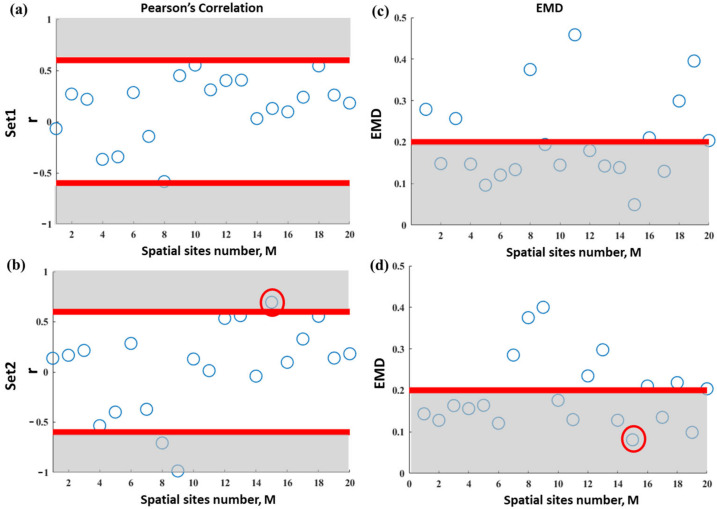
Pearson’s correlation (**a,b**) and EMD (**c,d**) on DF and MSF results for different spatial sites M in Patient1 Set 1 and Set 2 with ${\bar{BP}}^{th}$ = 15 Hz. Thresholds of r^th^ = ±0.6 and EMD^th^ = 0.2 (indicated as red lines), from a previous study [19], were chosen to distinguish the strong correlation relationship for Pearson’s correlation and EMD. Shadow area represents a highly correlated relationship for two methods. No common spatial sites were identified using Pearson’s correlation and EMD measures evaluated from Set 1. However, substrate spatial site 15 (red circle) shows high-correlation relationships between DF and MSF analyses in both Pearson’s correlation and EMD evaluated from Set 2. According to previous research, the substrate site (#15 here) is believed to be the potential ablation target.

Figure 6 shows the results of Pearson’s correlation and EMD, respectively, calculated using Equations (3) and (4) for all M = 20 different spatial sites for Set 1 and Set 2 of Patient 1 with ${\bar{BP}}^{th}$ = 15 Hz. A threshold of r^th^ = ± 0.6 and EMD^th^ = 0.2 (indicated as red lines) from a previous study [21] was chosen to distinguish the strong relationship for the Pearson’s correlation and EMD, respectively, in Figure 6a,b. The points in the grey area represent a strong correlation (r > 0.6 or r < −0.6 and EMD < 0.2), indicating high similarity between DF and MSF.

No spatial sites with significant Pearson’s correlation and EMD were identified in Set 1, i.e., none of the M = 20 spatial sites were highly correlated in both Pearson’s correlation and EMD implementations. However, spatial site 15 in Set 2 (red circle in Figure 6c,d) shows a high correlation between DF and MSF analyses in both Pearson’s correlation and EMD, thus indicating a possible presence of abnormal electrical activity at this site. For further use, we define these sites, in which both Pearson’s and EMD correlation methods show significant results, as “substrate” sites. Therefore, the removal of noisy and contact-loss leads benefits DF and MSF analysis in terms of the consistency between the two analyses. More similar correlation analyses were carried out, as illustrated in the following section.

#### 3.2.2. Correlation Analysis on Eight Patients with Known AF Ablation Outcomes

Table 2 lists the clinical outcomes of ablation (termination or no termination) and the number of substrate sites for eight of the patients. Note that, in the two patients with no AF termination from Table 2, substrate sites were identified in Set 2 using ${\bar{BP}}^{th}$ = 15 Hz, whereas in three of the four AF-terminated patients, no substrate sites were observed. When the same analysis was repeated using Set 1 with ${\bar{BP}}^{th}$ = 15 Hz, no substrate sites were identified in any patients. To further illustrate the necessity of filtering, we performed the same Pearson’s correlation and EMD analysis on eight of the patients with Set 2 without any filtering. No substrate sites were identified in any patients, as seen in Table 2’s third column. Thus, the removal of noisy and contact-loss leads plus BP filtering with efficient range during preprocessing is essential because it benefits DF and MSF analysis in terms of consistently identifying sites with abnormal electrical activities. Furthermore, the abnormal electrical sites identified in the no termination patients may have the potential to be target sites of ablation and may lead to AF.

## 4. Discussion

In this research, a data-driven framework for preprocessing was determined using frequency analysis of iEGMs from nine different patients with AF. The main results were summarized as follows: (1) the performance of both ideal and fifth-order IIR Butterworth BP filters are similar. (2) The upper bound of the bandpass filter with ${\bar{BP}}^{th}$ = 15 Hz is essential during the frequency analysis of iEGMs. (3) Abnormal electrical sites were identifiable only after the removal of noisy leads and applying the BP filter within an efficient range. (3) Abnormal electrical sites were not identified in any AF termination cases, thus showing potential to be target sites for ablation.

### 4.1. Importance of Removal of Contact-Loss Leads

In catheter mapping, the loss of contact of electrodes causes the iEGMs to return reduced or zero information content. This leads to erroneous measurements during iEGM analysis. Noise removal is a crucial component in the preprocessing of EGMs. In previous work, noise removal was carried out using a third-order IIR Butterworth filter and the core of the rotor was identified using simulated iEGM signals during AF using MSF [23]. In another work, MSF analysis was carried out on iEGM signals to identify the presence of abnormal electrical activity during AF, where the removal of noisy signals was carried out by eliminating any clinical iEGM signals with a low SNR [30]. Similarly, in our current work, we made a signal selection wherein noisy signals were removed from the analysis when the SNR was poor. Moreover, in this study, we showed, using the correlation and EMD approaches, that the removal of noisy and contact-loss electrodes improves the identification of target sites in patients with no AF termination. In another previous study, the contact-loss electrodes were removed from the analysis [30] to identify new targets for AF ablation. Thus, the removal of contact-loss leads is a necessary and essential step in the preprocessing of iEGMs.

### 4.2. Importance of Efficient ${\bar{BP}}^{th}$ Identification

In current mapping systems, frequency analysis is performed on iEGMs to obtain DF maps. However, in this work, we have shown that DF, which is widely used in clinical applications, is very sensitive to the utilized bandpass filter. The organization index (OI) of DF can also be affected by the ${\bar{BP}}^{th}$; thus, an improper ${\bar{BP}}^{th}$ may lead to a badly pronounced DF. As we increased the ${\bar{BP}}^{th}$ of the filter, the range of values observed in different sites of the patients varied significantly and the mean of MSF and DF also increased accordingly. This led to the inaccurate identification of possible substrate sites. Therefore, it is essential to identify and use an efficient ${\bar{BP}}^{th}$ during the preprocessing of iEGMs. In a previous study, using frequency analysis [19], it was shown that localized high frequency regions exist in patients with AF. Furthermore, such sites have also been shown to increase AF cycle length. Thus, the frequency spectrum across atria in patients with AF is nonuniform. Therefore, in some cases, the individual values of DF and MSF are very different from the mean values across atria. In this study, we used the mean values of DF and MSF to present the influence of the BP filter alone. Individual sites were analyzed separately during AF outcome correlation analysis and the results were shown to identify sites of interest.

### 4.3. Limitations

The present study has several limitations, as follows. Currently, this research focuses on frequency analysis of clinical bipolar atrial iEGMs and is limited to certain analyses (MSF and DF), and filters (ideal and IIR Butterworth filters). More works including but not limited to tuning up need to be carried out when considering other cases such as ventricle iEGMs, different analyses (multiscale entropy, kurtosis), digital BP filters, etc. In this study, datasets of iEGMs were collected using sequential mapping by PENTARAY Catheter. Previously [23], we performed numerical simulations and validated our mapping approaches using both PENTARAY and GRID catheters. The performance of our approaches on the stimulated iEGMs for rotor core identification was comparable between these two types of sequential mapping catheters. Thus, we believe that the results and conclusions of the current study can be extended to various types of sequential mapping catheters. Indeed, we have never utilized the information regarding different electrode configurations in our framework. However, for some other very different scenarios such as a 64-pole basket catheter with simultaneous mapping, this framework needs to be further investigated.

Since we are working on clinical atrial iEGMs, the ground truth of DF and MSF is unknown. However, the application of frequency analysis of simulated iEGMs from human atria models was recently carried out and the feasibility of identifying abnormal electrical activities was proven [23]. Regarding clinical atrial iEGMs [6], the DF and MSF analyses were able to detect abnormal sites. In this study, we removed all iEGMs with an extremely low amplitude, long gap, high noise corruption, loss of contact, low SNR, and white noise, thus retaining the reliability to apply DF and MSF analysis.

In this study, only an ideal BP and a fifth-order IIR Butterworth filter were implemented, and frequency analysis results stayed similar in our scenarios. In previous studies [14,15,16,17,18,19,20,21,22,23], the IIR Butterworth filter, as a very commonly used BP filter, was implemented in several applications to iEGMs. Thus, our study provides the desirable range of 3–15 Hz for iEGM application, which is the most important parameter when applying BP filtering in a practical sense. Another limitation of our study is the relatively small number of patients (*n* = 9) and limited number of iEGM recording sites. Our reported most efficient filtering range of 3–15 Hz might be insignificantly altered in the case of more datasets. We, however, do not expect any significant changes to our reported values.

In addition, all patients underwent only pulmonary vein (PV) isolation. When the highly correlated sites are outside the PV region, and only PV isolation is performed, AF should not be terminated. However, due to the insufficient information on the spatial location of the recorded clinical iEGM dataset, the identified regions in the no termination cases were not correlated to the anatomy of the heart. Therefore, further research should be conducted with a large number of patients and with prior knowledge of precise spatial sites. In the current study, we did not perform any AF ablation after identification of the substrate sites due to the retrospective nature of the available data. Only prospective AF ablation studies, where iEGMs are analyzed and compared pre- and post-ablation, can provide a direct confirmation of the actual substrate site. However, this current study can still provide us a good prospective and suggestions for the potential target of AF ablation.

## 5. Conclusions

Our research has identified that a 3–15 Hz preprocessing BP filter range is the most efficient for iEGM frequency analysis. While increasing the ${\bar{BP}}^{th}$ from 10 Hz to 15 Hz, some frequency components shifted from the lower frequency cluster and stayed, which can be important in patients with AF. The results of the Pearson’s correlation and EMD indicate that removing noisy and contact-loss leads helps obtain higher correlation and consistency between frequency analysis techniques. Thus, it is necessary to remove noisy and contact-loss leads and apply efficient BP filtering for clinically recorded iEGM datasets prior to analysis. These results help in building a data-driven preprocessing framework of well-defined BP filters for data analysis using clinically recorded iEGMs.

## Figures and Tables

**Table 1:** Summary of Set 1—all iEGMs and Set 2—clean iEGMs dataset for 9 patients. M: number of spatial sites. N: number of electrodes. Total_1_: total number of iEGMs in Set1. Total_2_: total number of iEGMs in Set2.

	Set 1: All iEGMs			Set 2: Clean iEGMs			AF (Termination or No Termination)
	M	N	Total_1_	M	N	Total_2_	
Patient 1	20	10	200	20	5 to 10	141	Unknown
Patient 2	18	10	180	18	5 to 10	180	No
Patient 3	20	10	200	20	5 to 10	146	Yes
Patient 4	16	10	160	16	5 to 10	121	No
Patient 5	25	10	250	22	5 to 10	115	Yes
Patient 6	19	10	190	19	5 to 10	190	Yes
Patient 7	25	10	250	24	5 to 10	211	Yes
Patient 8	20	10	200	19	5 to 10	166	No
Patient 9	20	10	200	20	5 to 10	136	No

**Table 2:** Summary of Set 1—all iEGMs, Set 2—clean iEGMs dataset with ${\bar{BP}}^{th}$ = 15 Hz; and Set 2—clean iEGMs dataset without any filtering for nine patients.

	$\mathrm{Number}\;\mathrm{of}\;\mathrm{Substrate}\;\mathrm{Sites}\;\mathrm{in}\;\mathrm{Set}\;1\;({\bar{BP}}^{th}=15\;Hz)$	$\mathrm{Number}\;\mathrm{of}\;\mathrm{Substrate}\;\mathrm{Sites}\;\mathrm{in}\;\mathrm{Set}\;2\;({\bar{BP}}^{th}=15\;Hz)$	Number of Substrate Sites in Set 2 (No Filtering)	AF (Termination or No Termination)
Patient 2	None	2	None	No
Patient 3	None	4	None	Yes
Patient 4	None	1	None	No
Patient 5	None	None	None	Yes
Patient 6	None	None	None	Yes
Patient 7	None	None	None	Yes
Patient 8	None	6	None	No
Patient 9	None	3	None	No

## Supplementary Materials

The following supporting information can be downloaded at: https://www.mdpi.com/article/10.3390/e25020332/s1, Table S1: Summary of Frequency Analysis Techniques, Figure S1: DF and MSF values and their distributions calculated from iEGMs of Patient 1 Set 2, from 20 spatial sites after applying ideal BP filter with various ${\bar{BP}}^{th}$ and distributions as a function of ${\bar{BP}}^{th}$; Figure S2: DF and MSF values and their distributions calculated from iEGMs of Patient 1 Set 2, from 20 spatial sites after applying IIR Butterworth BP filter with various ${\bar{BP}}^{th}$ and distributions as a function of ${\bar{BP}}^{th}$; Figure S3: DBSCAN clustering result of Patient 1 Set 2 represented as $\bar{DF}$ vs $\bar{MSF}$ dependence for ideal BP filter ${\bar{BP}}^{th\;}$; Figure S4: DBSCAN clustering result of Patient 1 Set 2 represented as $\bar{DF}$ vs $\bar{MSF}$ dependence for IIR Butterworth ${\bar{BP}}^{th\;}$.
