# Supplementary material for: A Data-Driven Preprocessing Framework for Atrial Fibrillation Intracardiac Electrocardiogram Analysis
Source: Entropy (Basel). 2023 Feb 10;25(2):332. doi: 10.3390/e25020332 (PMC9955244; doi:10.3390/e25020332)
Supplement: Supplementary file 1 [file entropy-25-00332-s001.zip › entropy-2053388-supplementary.pdf]

Supplemental Material

1. *Frequency and Temporal Signal Analysis Techniques:*

The multiscale frequency (MSF) approach was previously developed in our lab and is implemented on the intracardiac electrograms (EGM) along with the traditional dominant frequency (DF) approach. The MSF is a frequency-based approach where multiple instantaneous local frequencies are averaged and are more efficient than the DF approach, where only the frequency with the maximum magnitude was considered. Also, the MSF has been shown to discriminate between the AF and NSR using surface ECG.

Information on the frequency analysis approaches is listed in Table S1 provided below. All techniques were implemented on the electrogram signals in our work.

Method	Equation	Variable Definition	Description
**Dominant Frequency (DF)**	$DF = f(\max(X))$	*f: Frequency spectrum*<br>*X: magnitude FFT of the input time series x(t)*<br>*DF: Dominant Frequency*	*Calculates the dominant frequency.*
**Multiscale Frequency (MSF)**	$MSF = \rho_0 \left[\sum_{i=1}^{N-1} q_i\right]^{-1} \sum_{i=1}^{N-1} 2^{i+0.5} q_{i+1}$	q_i: *Output of the ith log-Gabor Filter*<br><br>ρ_0: *center frequency of the first log-Gabor filter*<br><br>*N: the total number of samples*	*Calculates the instantaneous frequency using the signal spectrum of the electrograms (time series).*

Table S.1 Summary of Frequency Analysis Techniques

2. DF and MSF calculation and clustering under ideal and Butterworth bandpass filter

Here, we are giving another example of MSF and DF values calculated from iEGMs of Patient 1 Set 2, from 20 spatial sites after applying ideal BP filter (Fig.S1) or Butterworth filter Fig.S2 with various $\overline{BP}^{th}$, ranging from 10 Hz to 30 Hz. Fig. S3 panel (a) to (g) demonstrate one example of the formation of clusters as $\overline{BP}^{th}$ increases on Patient1 Set2, using ideal bandpass filter. Similar result can be also seen in Fig.S4 with the fifth order Butterworth filter. The number of cluster and DI were summarized in Fig.3. Note that $\overline{BP}^{th}$ = 15 Hz had maximum DI over all $\overline{BP}^{th}$ values, which indicates that the clusters are compact.

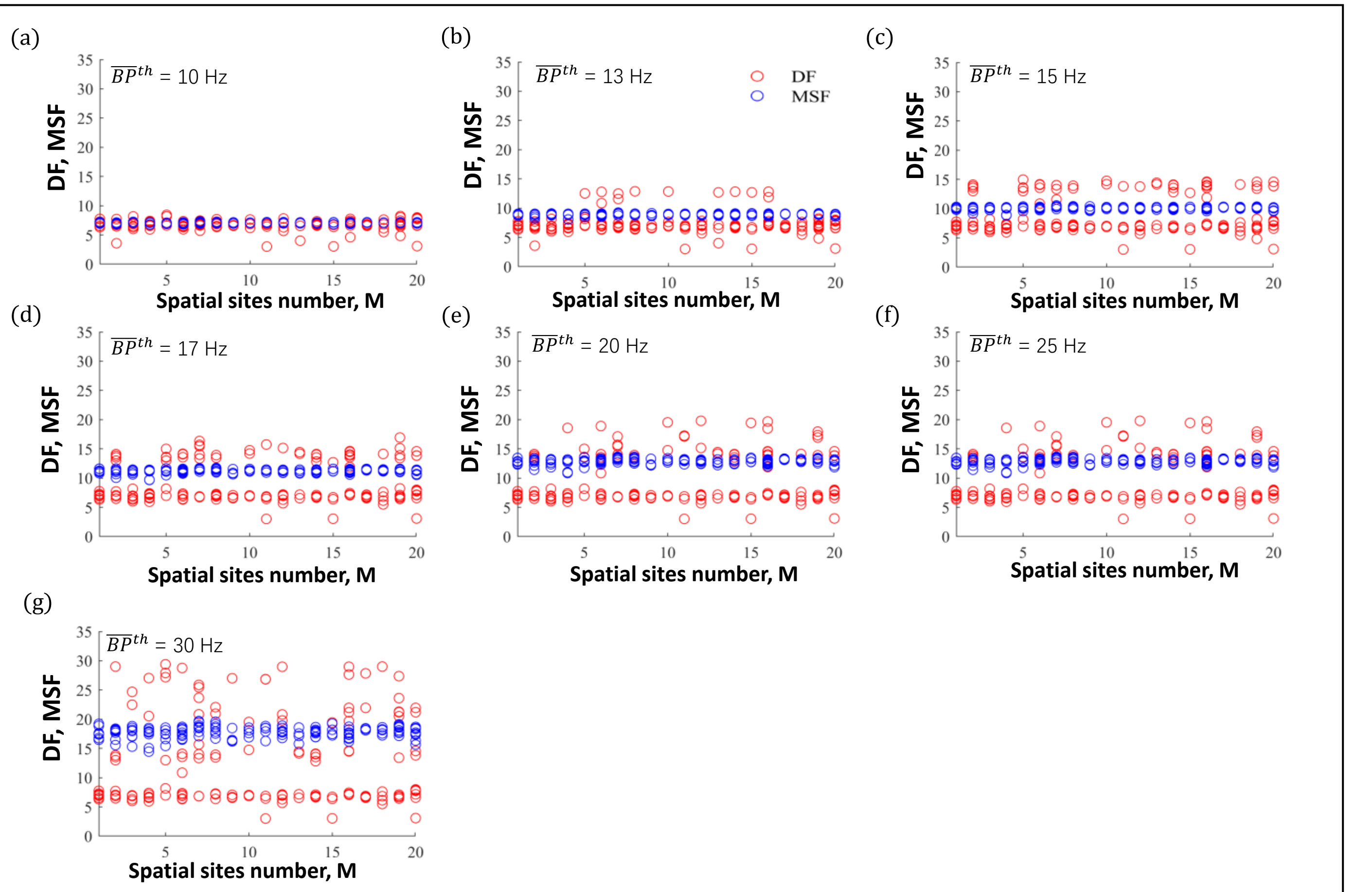

Figure S1. DF and MSF values and their distributions calculated from iEGMs of Patient 1 Set 2, from 20 spatial sites after applying ideal BP filter with various $\overline{BP}^{th}$and distributions as a function of $\overline{BP}^{th}$: **(a)** 10 Hz, **(b)** 13 Hz, **(c)** 15 Hz, **(d)** 17 Hz, **(e)** 20 Hz, **(f)** 25 Hz, **(g)** 30 Hz.

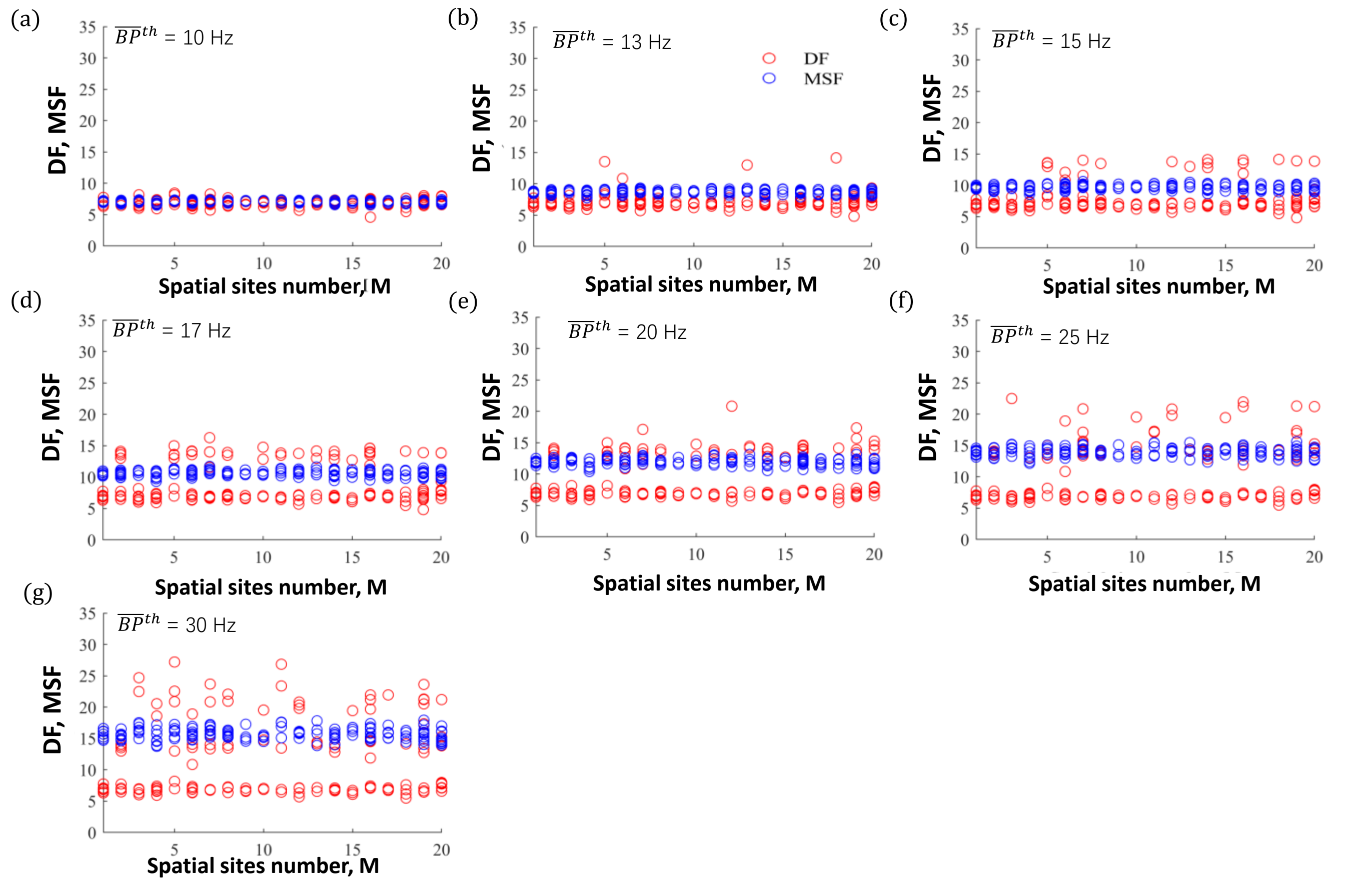

Figure S2. DF and MSF values and their distributions calculated from iEGMs of Patient 1 Set 2, from 20 spatial sites after applying IIR Butterworth BP filter with various $\overline{BP}^{th}$ and distributions as a function of $\overline{BP}^{th}$: **(a)** 10 Hz, **(b)** 13 Hz, **(c)** 15 Hz, **(d)** 17 Hz, **(e)** 20 Hz, **(f)** 25 Hz, **(g)** 30 Hz.

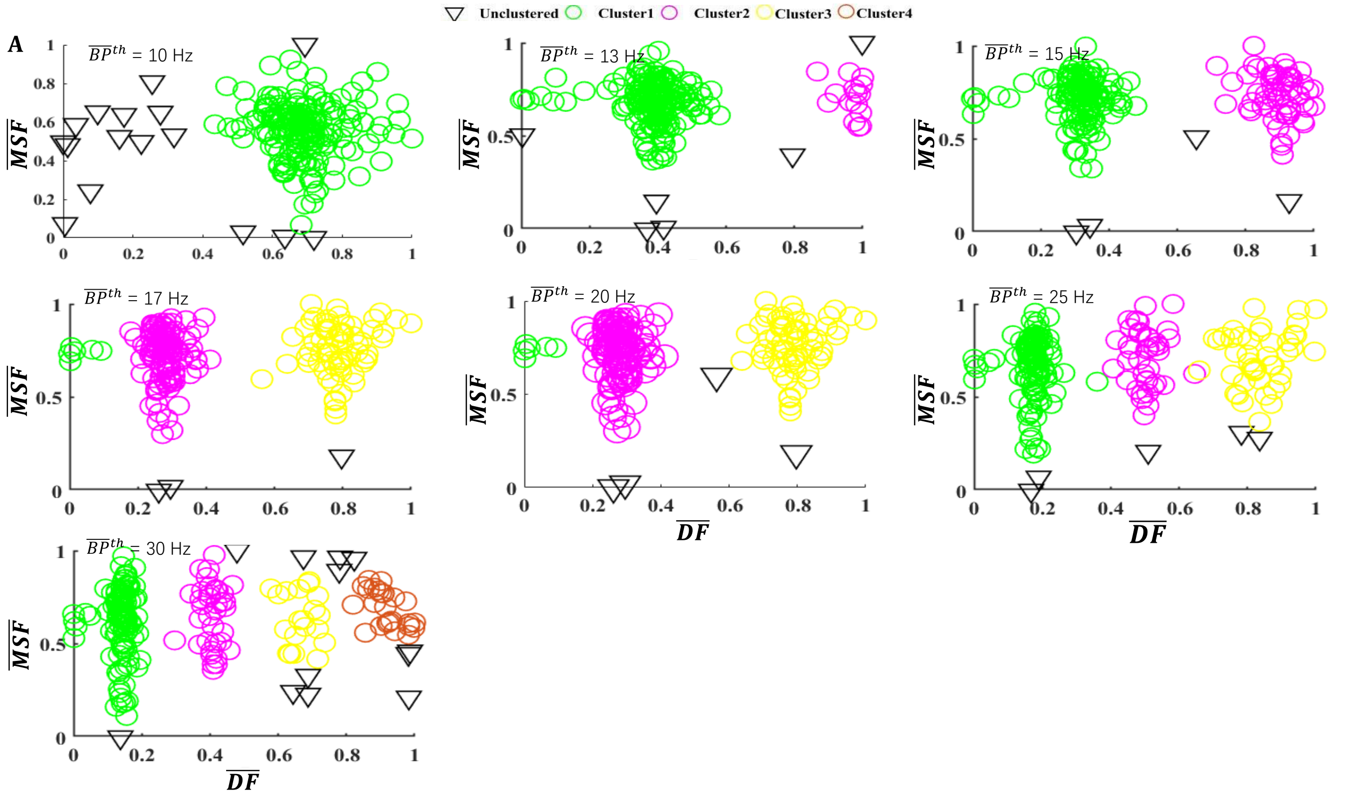

Figure S3. DBSCAN clustering result of Patient 1 Set 2 represented as $\overline{DF}$ vs $\overline{MSF}$ dependence for ideal BP filter $\overline{BP}^{th}$: **(a)** 10 Hz, **(b)** 13 Hz, **(c)** 15 Hz, **(d)** 17Hz, **(e)** 25 Hz, **(f)** 25 Hz, **(g)** 30 Hz.

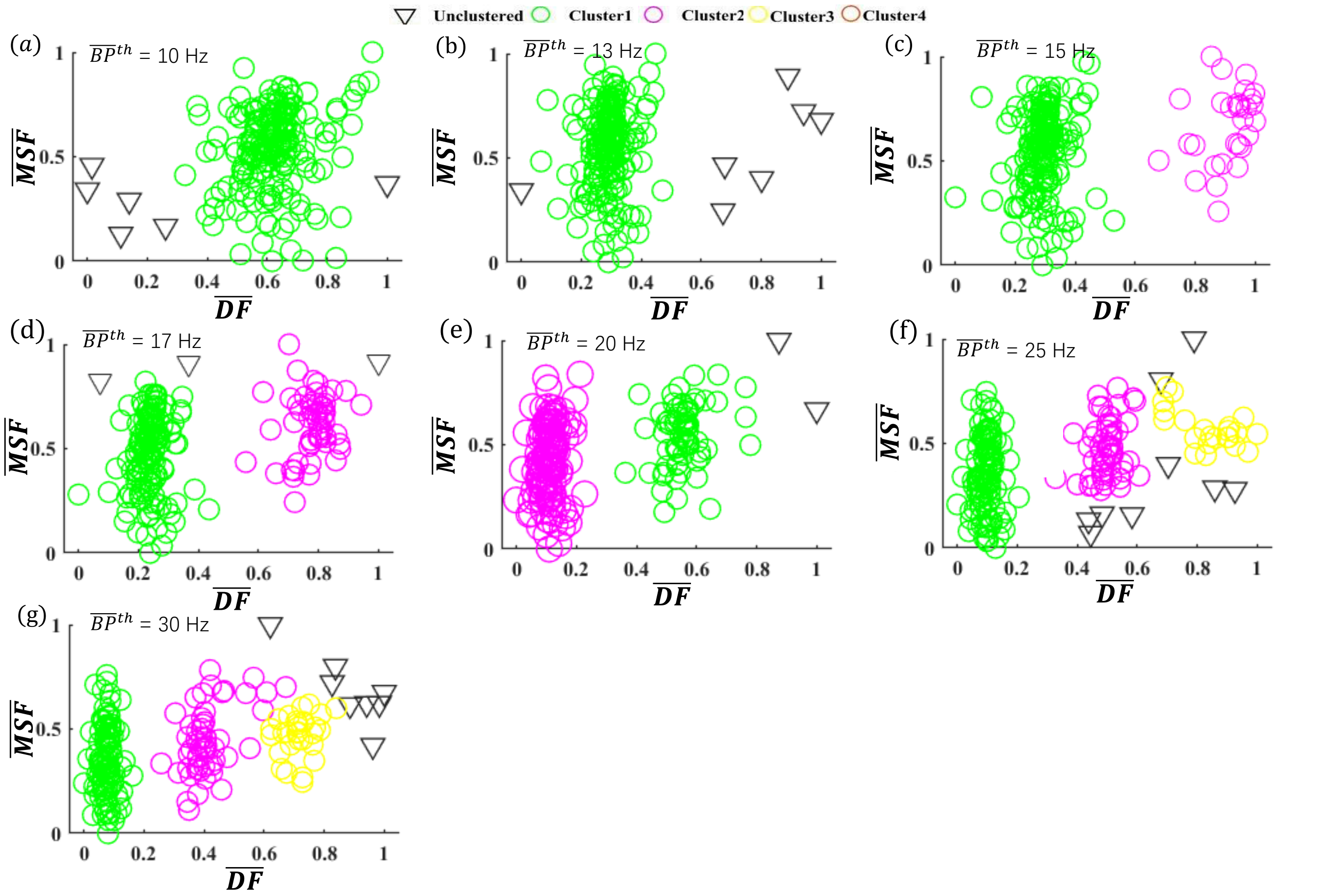

Figure S4. DBSCAN clustering result of Patient 1 Set 2 represented as $\overline{DF}$ vs $\overline{MSF}$ dependence for IIR Butterworth $\overline{BP}^{th}$: **(a)** 10 Hz, **(b)** 13 Hz, **(c)** 15 Hz, **(d)** 17Hz, **(e)** 25 Hz, **(f)** 25 Hz, **(g)** 30 Hz.
